# cFLIP critically modulates apoptotic resistance in epithelial-to-mesenchymal transition

**DOI:** 10.18632/oncotarget.19557

**Published:** 2017-07-25

**Authors:** Chandrasekhar Padmanabhan, Eric J. Rellinger, Jing Zhu, Hanbing An, Luke G. Woodbury, Dai H. Chung, Alex G. Waterson, Craig W. Lindsley, Anna L. Means, R. Daniel Beauchamp

**Affiliations:** ^1^ Section of Surgical Sciences, Vanderbilt University Medical Center, Nashville TN, 37232, USA; ^2^ Department of Surgery, Vanderbilt University Medical Center, Nashville TN, 37232, USA; ^3^ Department of Pediatric Surgery, Vanderbilt University Medical Center, Nashville TN, 37232, USA; ^4^ Department of Cancer Biology, Vanderbilt University School of Medicine, Nashville TN 37232, USA; ^5^ Department of Pharmacology, Vanderbilt University School of Medicine, Nashville TN, 37232, USA; ^6^ Vanderbilt Center for Neuroscience Drug Discovery, Vanderbilt University Medical Center, Nashville TN, 37232, USA; ^7^ Vanderbilt Institute of Chemical Biology, Vanderbilt University, Nashville TN, 37232, USA; ^8^ Department of Cell and Developmental Biology, Vanderbilt University School of Medicine, Nashville TN, 37232, USA; ^9^ The Vanderbilt-Ingram Cancer Center, Vanderbilt University Medical Center, Nashville TN, 37232, USA

**Keywords:** EMT, apoptosis, cFLIP, TRAIL, isoxazole

## Abstract

Epithelial cancers (carcinomas) comprise the top four causes of cancer-related deaths in the United States. While overall survival has been steadily improving, therapy-resistant disease continues to present a major therapeutic challenge. Carcinomas often exploit the normal developmental program, epithelial-to-mesenchymal transition (EMT), to gain a mesenchymal phenotype associated with increased invasiveness and resistance to apoptosis. We have previously shown that an isoxazole-based small molecule, ML327, partially reverses TGF-β-induced EMT in an immortalized mouse mammary epithelial cell line. Herein, we demonstrate that ML327 reverses much of the EMT gene expression program in cultured carcinoma cell lines. The reversal of EMT sensitizes these cancer cells to the apoptosis-inducing ligand TRAIL. This sensitization is independent of E-cadherin expression and rather relies on the downregulation of a major anti-apoptotic protein, cFLIP_S_. Loss of cFLIP_S_ is sufficient to overcome resistance to TRAIL and exogenous overexpression of cFLIP_S_ restores resistance to TRAIL-induced apoptosis despite EMT reversal with ML327. In summary, we have utilized an isoxazole-based small molecule that partially reverses EMT in carcinoma cells to demonstrate that cFLIP_S_ critically regulates the apoptosis resistance phenotype associated with EMT.

## INTRODUCTION

Epithelial cancers (carcinomas) comprise the top four causes of cancer related deaths in the United States [1]. Overall survival for these malignancies has been steadily improving over the past several decades largely due to improved screening along with aggressive medical and surgical intervention. Despite these improvements, a major therapeutic challenge persists because cancer progression is usually associated with treatment resistance. Cytotoxic chemotherapy induces apoptosis in susceptible, rapidly proliferating, cancer cells. Certain populations of cancer cells, however, obtain a stem-cell-like phenotype, divide slowly, resist apoptosis, and are thought to drive progression and recurrence despite aggressive medical therapy [2].

Epithelial-to-mesenchymal transition (EMT) is a major cellular reprogramming of carcinomas that not only results in a mesenchymal phenotype, characterized by repression of E-cadherin expression and increased cancer cell invasiveness, but also results in a chemotherapeutic-resistant state [3]. Cells that have undergone EMT also acquire a cancer stem cell-like phenotype [4]. It has been proposed that these cancer stem cells evade apoptosis and ultimately lead to disease progression or recurrence [2, 5]. The potential to reverse EMT and re-sensitize cancer cells to apoptosis-inducing agents represents a novel strategy for the treatment of carcinomas.

Therapeutic application of the tumor necrosis factor-related apoptosis-inducing ligand (TRAIL, also known as APO2L or TNFSF10), a death receptor ligand, is especially appealing as it selectively induces apoptosis in cancer cells both *in vitro* and *in vivo* with minimal toxicity toward non-cancerous cells [6, 7]. While TRAIL and other death receptor agonists have been found safe and well tolerated in phase 1 and phase 2 clinical trials, these agents have not demonstrated any clinically significant anti-tumor effect when compared to standard therapy alone [8]. This failure to progress in clinical trials is likely due to pre-existing resistance to TRAIL in some cancer cells, and the rapid acquisition of resistance in others; however, the exact mechanism of this resistance is inadequately understood.

Anti-apoptotic proteins have been implicated in TRAIL resistance as these are often overexpressed in cancers and promote tumor progression and treatment failure [9, 10]. One such protein, the cellular FLICE-like inhibitory protein (cFLIP, also known as CASP8 and FADD-like apoptosis regulator, or CFLAR), is a potent anti-apoptotic protein known to negatively regulate TRAIL-induced apoptosis. cFLIP is expressed primarily as two dominant splice variants, cFLIP Long (cFLIP_L_) and cFLIP Short (cFLIP_S_) [10]. While cFLIP_L_ has been shown to be pro-apoptotic at physiologic levels, and not anti-apoptotic as previously thought [11–13], cFLIP_S_ is clearly an anti-apoptotic protein. Structurally homologous to caspase 8, cFLIP_S_ lacks inherent caspase catalytic activity and prevents release of active caspase 8 from the death-inducing signaling complex (DISC). Thus, cFLIP_S_ triggers cells to activate pro-survival signaling pathways in response to TRAIL rather than pro-apoptotic pathways [14].

EMT also plays a major role in TRAIL resistance [15]. Indeed, it has been proposed that E-cadherin expression is necessary for apoptosis induction by TRAIL [16]. We previously characterized and reported our discovery of an isoxazole-based small molecule probe, ML327, that de-represses E-cadherin expression and partially reverses the EMT phenotype [17, 18]. In the current report, we demonstrate that EMT reversal by ML327 is accompanied by an augmented response to the TRAIL ligand in carcinoma cells that is independent of E-cadherin expression. EMT reversal with ML327 resulted in a consistent downregulation of cFLIP_S_ expression across a variety of cancer cell lines and our data support this downregulation of cFLIP_S_ as the mechanism by which ML327 sensitizes carcinomas to TRAIL-induced apoptosis.

## RESULTS

### ML327 partially reverses EMT in carcinoma cells

**Figure 1:**
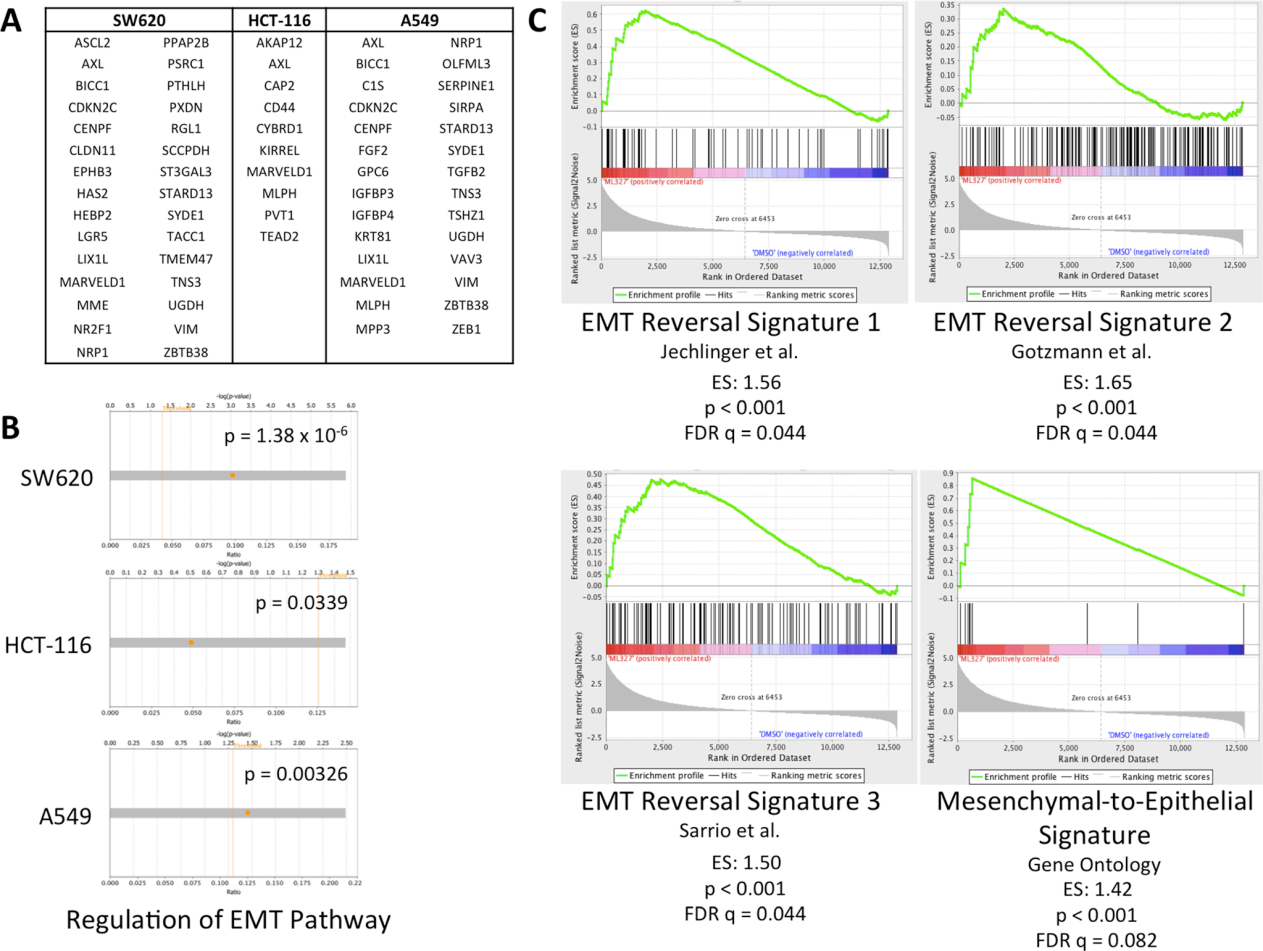
ML327 partially reverses EMT in carcinoma cells (**A**) Table of EMT and stem markers upregulated in EMT that are downregulated (FDR < 0.05) across 3 cell lines after 24-hour ML327 treatment (10 μM). (**B**) Core expression analysis of RNAseq data (log2FC > 2, FDR < 0.0001) from SW620, HCT-116, and A549 cells implicated “Regulation of the EMT Pathway” as a top canonical organismal growth and development pathways. Fisher Exact Test *p*-values of significance indicated. (**C**) Gene set enrichment analysis of RNAseq data from SW620 cells treated with ML327 (10 μM) or vehicle for 24 hours. Results demonstrate positive enrichment of EMT reversal signatures with *p* < 0.001 and FDR *q* < 0.09.

Our previous work demonstrated a partial reversal of TGF-β-induced EMT with ML327 at a 10 μM concentration in an immortalized mouse mammary epithelial cell line as well as upregulation of E-cadherin in multiple cell lines [17]. We proceeded to test whether ML327 broadly regulates markers of EMT in several carcinoma cell lines independently of TGF-β treatment and therefore performed RNA sequencing (RNAseq) on HCT-116, SW620, and A549 cancer cell lines treated with 10 μM ML327 (or vehicle control) for 24 hours (Supplementary Tables 1–3). Sequencing data demonstrated similar gene expression changes across all 3 cancer cell lines with 730 commonly upregulated genes and 37 commonly downregulated genes (Supplementary Table 4). EMT and stem cell markers that are typically upregulated during EMT were downregulated after ML327 treatment (Figure 1A). Core expression analysis of the RNAseq data using Ingenuity Pathway Analysis (IPA) implicated “Regulation of the Epithelial-to-Mesenchymal Transition Pathway” as one of the top organismal growth and development pathways in all 3 cancer cell lines (Figure 1B, Supplementary Figure 1A). We further assessed the RNAseq findings using gene set enrichment analysis (GSEA) and found positive enrichment of previously published EMT reversal signatures [19–21] as well as Gene Ontology (GO version 5.2) adherens junction functioning signatures [22, 23] (Figure 1C, Supplementary Figure 1B). Taken together, these data demonstrate that ML327 treatment partially reverses EMT in carcinoma cells.

### EMT reversal sensitizes carcinoma cells to TRAIL-induced apoptosis

**Figure 2:**
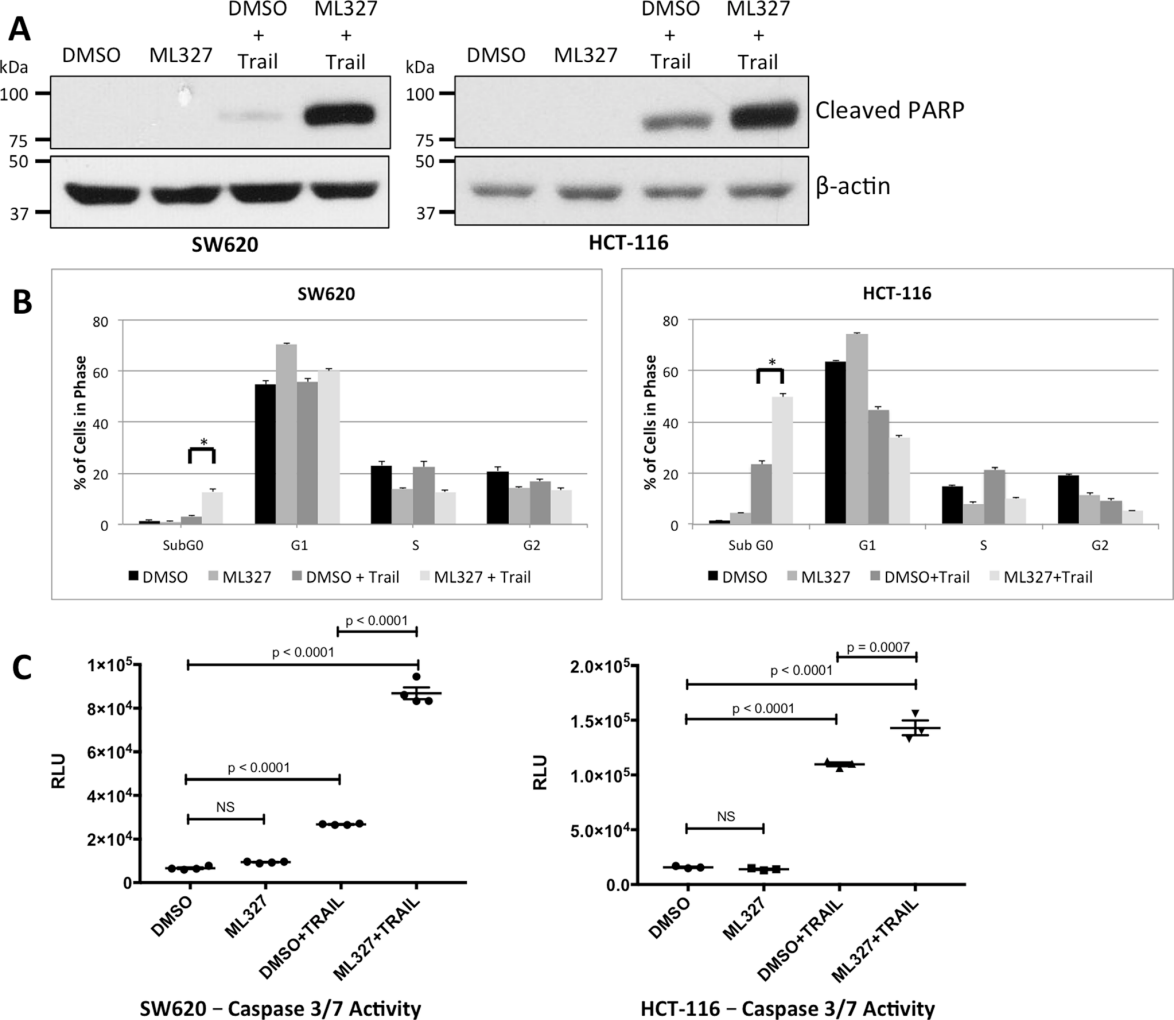
EMT reversal with ML327 sensitizes carcinoma cells to the apoptosis-inducing ligand TRAIL (**A**) Western blot analysis of two carcinoma cell lines that were treated with 10 μM ML327 (or vehicle) for 24 hours followed by TRAIL (SW620: 100 ng/mL; HCT-116: 50 ng/mL). ML327 pre-treatment resulted in increased PARP cleavage with TRAIL in both cell lines as compared to vehicle pre-treatment. ML327 alone did not cause any PARP cleavage. (**B**) Under similar treatment conditions, cells were fixed, stained with propidium iodide, and analyzed by FACS for cell-cycle composition. ML327 pre-treatment prior to TRAIL demonstrated an increased percentage of cells in the Sub G_0_ population as compared to vehicle pre-treatment (SW620: *p* < 0.01; HCT-116: *p* < 0.01). ML327 alone did not increase the percentage of cells in the Sub G_0_ population. Data are mean ± SEM from *n* = 3 biologic replicates. Two-way ANOVA was performed to analyze all means. (**C**) Caspase 3/7 activation was assessed via the luminescence-based Caspase-Glo^®^ 3/7 Assay System under similar treatment conditions as in (A). ML327 pre-treatment followed by TRAIL for 4 hours resulted in increased caspase 3/7 activity, as measured by luminescence (RLU), when compared to vehicle pre-treatment in both SW620 cells (*p* < 0.0001) and HCT-116 cells (*p* = 0.0007). ML327 alone did not increased caspase 3/7 activity in either cell line. Data represented as mean ± SEM. Data points represent technical replicates. One-way ANOVA was performed to compare all means.

EMT is associated with resistance to therapy-induced apoptosis. As such, we tested whether partial EMT reversal with ML327 treatment would sensitize cancer cells to the apoptosis-inducing ligand, TRAIL. Three carcinoma cell lines were pre-treated with 10 μM ML327 for 24 hours and then TRAIL for 4 hours. ML327 pre-treatment alone did not result in poly ADP-ribose polymerase (PARP) cleavage. TRAIL treatment alone (after vehicle pre-treatment) resulted in low to moderate PARP cleavage, but the addition of TRAIL after ML327 pre-treatment resulted in a marked increase in PARP cleavage by 4 hours after TRAIL addition (Figure 2A, Supplementary Figure 2A). ML327 pre-treatment followed by TRAIL also increased Annexin V binding to phosphatidylserine (PS) residues by 2 hours and persisted at 12 hours after TRAIL treatment (*p* < 0.0001) (Supplementary Figure 2B). Caspase 8 cleavage was also increased by 4 hours after TRAIL addition (Supplementary Figure 2C). At later time points, too few viable cells remained to perform western blot analysis in the ML327+TRAIL treated cells (data not shown). We validated these findings with flow cytometric cell cycle analysis and luminescence-based caspase 3/7 activity assays under the same treatment conditions. Cell cycle analysis demonstrated an increased proportion of SW620 (*p* < 0.01) and HCT-116 (*p* < 0.01) cells in the Sub G_0_ phase with ML327 pre-treatment followed by TRAIL for 4 hours (Figure 2B). Caspase 3/7 activity was also increased with ML327 pre-treatment followed by TRAIL for 4 hours in SW620 (*p* < 0.0001) and HCT-116 (*p* = 0.0007) cells (Figure 2C). To determine whether ML327 sensitized a non-transformed cell line to TRAIL-induced apoptosis, we examined PARP cleavage and caspase 3/7 activity in immortalized, but not transformed, young-adult mouse colon epithelial cells (YAMC) [24] using similar treatment conditions as above. Interestingly, there was no PARP cleavage with TRAIL alone or in combination with ML327 (Supplementary Figure 2D) and there was no difference in caspase 3/7 activity with ML327 pre-treatment (as compared to vehicle pre-treatment) followed by TRAIL (Supplementary Figure 2E). Taken together, these data suggest that partial EMT reversal with ML327 sensitizes carcinoma cells to TRAIL-induced apoptosis while sparing non-cancerous cells.

### TRAIL sensitization following EMT reversal is independent of E-Cadherin expression

**Figure 3:**
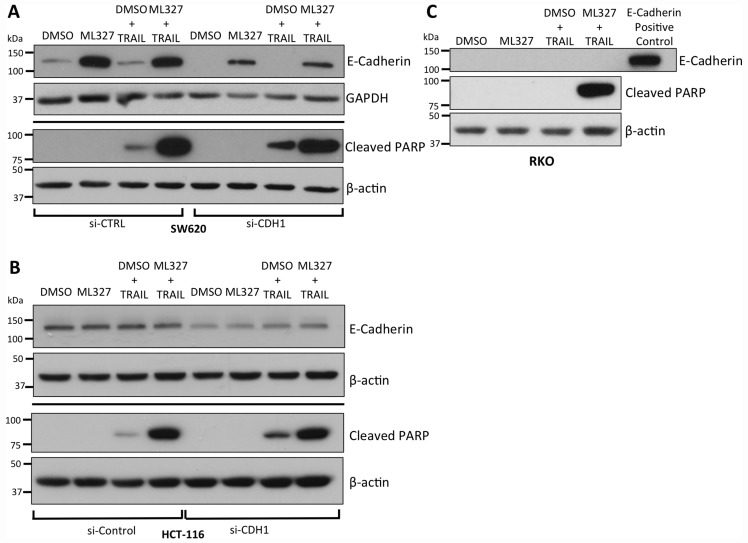
TRAIL sensitization by partial EMT reversal with ML327 is independent of E-cadherin expression (**A**) Western blot analysis of siRNA-mediated knockdown of E-cadherin in SW620 cells demonstrating decreased E-cadherin levels without a reduction PARP cleavage relative to β-actin controls after 10 μM ML327 pre-treatment (24 hours) followed by 100 ng/mL TRAIL (4 hours). (**B**) Similar analysis in HCT-116 cells, which constitutively express E-cadherin. Approximately 50% knockdown was achieved with no blunting of PARP cleavage after 10 μM ML327 pre-treatment (24 hours) followed by 50 ng/mL TRAIL (4 hours). (**C**) Western blot of RKO cells pre-treated with 10 μM ML327 for 24 hours followed by 500 ng/mL TRAIL for 4 hours, in which ML327 is unable to de-repress E-cadherin, demonstrates increased PARP cleavage compared to vehicle pre-treatment. E-cadherin positive control utilized was the same concentration of protein lysate derived from HCT-116 cells. All blots are representative of *n* = 3 biologic replicates.

A hallmark of EMT is loss of the adherens junction protein E-cadherin via transcriptional repression. E-cadherin has been shown to couple death receptors to the cytoskeleton and potentiate apoptotic signaling upon death ligand binding [16]. To test whether E-cadherin expression is necessary for TRAIL sensitization, we first performed small interfering RNA (siRNA) knockdown of E-cadherin in SW620 cells, in which E-cadherin is de-repressed after ML327 treatment, and HCT-116 cells, which constitutively express E-cadherin. In both cell lines, E-cadherin knockdown did not blunt enhanced PARP cleavage with ML327 pre-treatment followed by TRAIL (Figure 3A and 3B).

We next tested the effect of ML327 on TRAIL sensitization in RKO colon cancer cells in which ML327 is unable to de-repress E-cadherin expression due to promoter hypermethylation [25]. Despite the absence of E-cadherin expression, ML327 pre-treatment followed by TRAIL resulted in increased PARP cleavage (Figure 3C), increased caspase 3/7 activity (*p* = 0.0095) (Supplementary Figure 3A), increased cell percentage in the Sub G_0_ phase on cell cycle analysis (*p* < 0.001) (Supplementary Figure 3B), and increased Annexin V binding to PS residues (*p* < 0.0001) (Supplementary Figure 3C) as compared to vehicle pre-treatment. In addition, we pre-treated MDA-MB-231 breast cancer cells, which also harbor a hyper-methylated *CDH1* promoter, with ML327 followed by TRAIL and demonstrated increased percentage of cells in the Sub G_0_ cell population as compared to vehicle pre-treated cells (Supplementary Figure 3D). Taken together, these data demonstrate TRAIL sensitization after EMT reversal with ML327 is an E-cadherin-independent process.

### cFLIP_S_ modulates the apoptosis response in EMT

As E-cadherin de-repression was not required for ML327-induced TRAIL sensitization, we first assessed TRAIL receptor expression after ML327 treatment and found that ML327 had no effect on TRAIL-R1 and TRAIL-R2 expression (Supplementary Figure 4A). TRAIL receptor N-linked and O-linked glycosylation has also been reported to modulate TRAIL sensitivity in carcinoma cells [26, 27]. However, ML327 treatment did not cause a shift in band migration on immunoblot, nor were any additional bands present, suggesting no alteration in TRAIL receptor glycosylation state by ML327 (Supplementary Figure 4A).

**Figure 4:**
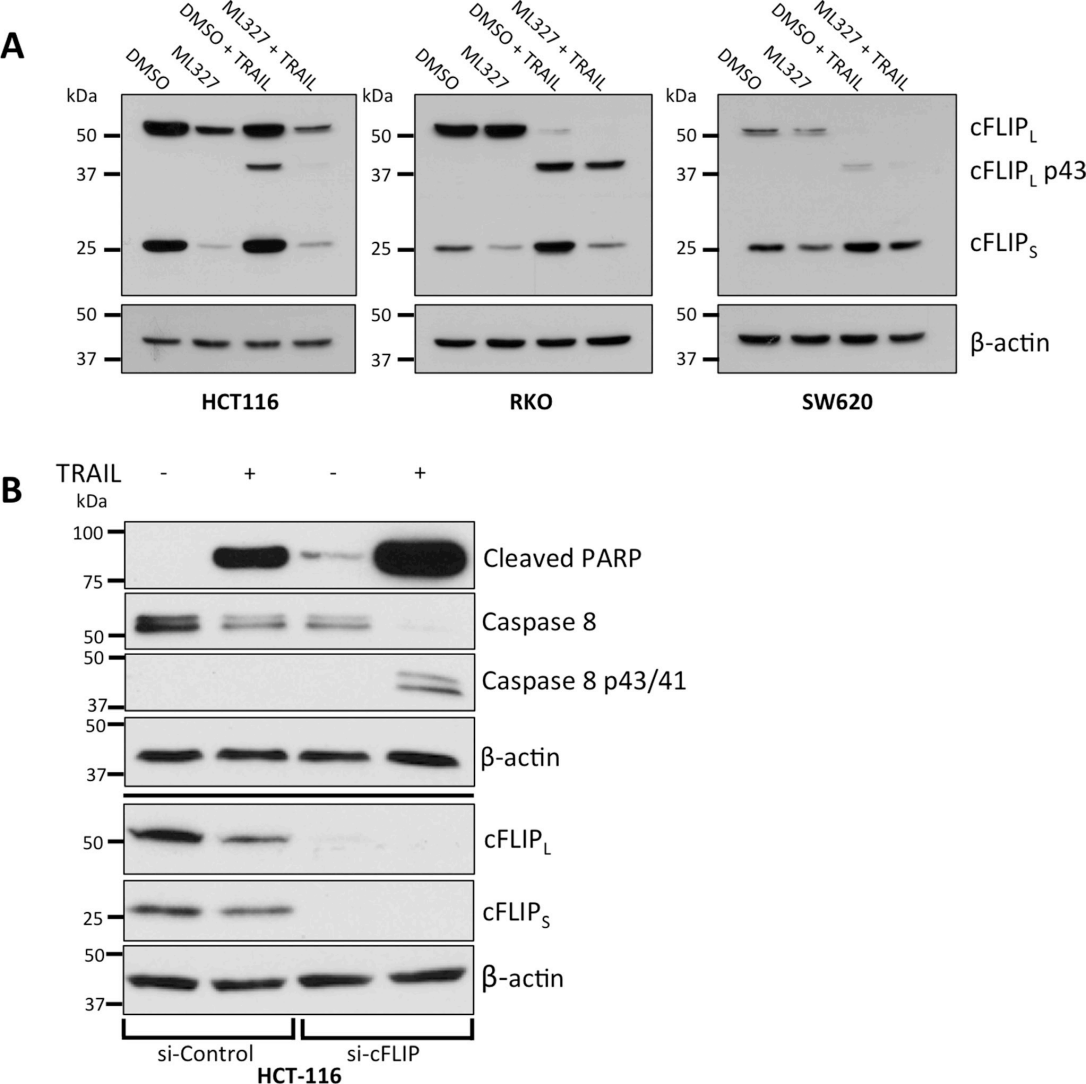
EMT reversal with ML327 is associated with a reduction in cFLIP_S_ and cFLIP_S_ loss is sufficient for TRAIL sensitization (**A**) Western blots demonstrating a consistent reduction of cFLIP_S_, but not cFLIP_L_, levels with and without TRAIL treatment in three carcinoma cell lines after 24-hour treatment of 10 μM ML327 as compared to vehicle treatment. In SW620 cells, TRAIL appears to upregulate cFLIP_S_ and this is blocked by ML327. Representative blots of *n* = 3 biologic replicates shown for all 3 cell lines. (**B**) Western blot of siRNA-mediated knockdown of cFLIP in HCT-116 cells treated with TRAIL (50 ng/mL). cFLIP knockdown was associated with increased caspase 8 and PARP cleavage. Representative blot of *n* = 3 biologic replicates shown.

We next performed a screen of several anti-apoptotic proteins including XIAP, cIAP1, cIAP2, Livin, Survivin, and cFLIP and observed consistent reduction of cFLIP_S_ protein levels after 24-hour ML327 treatment and no appreciable changes in the other proteins (Figure 4A, Supplementary Figure 4B, and data not shown). While cFLIP_S_ levels were consistently decreased by ML327 in SW620, HCT-116, A549 and RKO cells, cFLIP_L_ modulation was inconsistent, with decreased levels in HCT-116 cells and unchanged levels in SW620 and RKO cells (Figure 4A). cFLIP_L_ is thought to be pro-apoptotic at physiologic levels [11–13] whereas cFLIP_S_ has been implicated as a master anti-apoptotic regulator [28] and has been shown to inhibit CD95, TNF-α, and TRAIL-induced apoptosis [29–31]. We therefore focused our attention to cFLIP_S_.

To determine whether cFLIP_S_ loss was sufficient to sensitize cancer cells to TRAIL, we performed siRNA-mediated knockdown of cFLIP and demonstrated increased caspase 8 and PARP cleavage after treatment with TRAIL following loss of cFLIP expression (Figure 4B). Cells treated with control siRNA had less caspase 8 and PARP cleavage. The results of these experiments suggest that EMT reversal with ML327 leads to a reduction in cFLIP_S_ protein and that reduction of cFLIP_S_ is sufficient to sensitize cancer cells to TRAIL-induced apoptosis.

**Figure 5:**
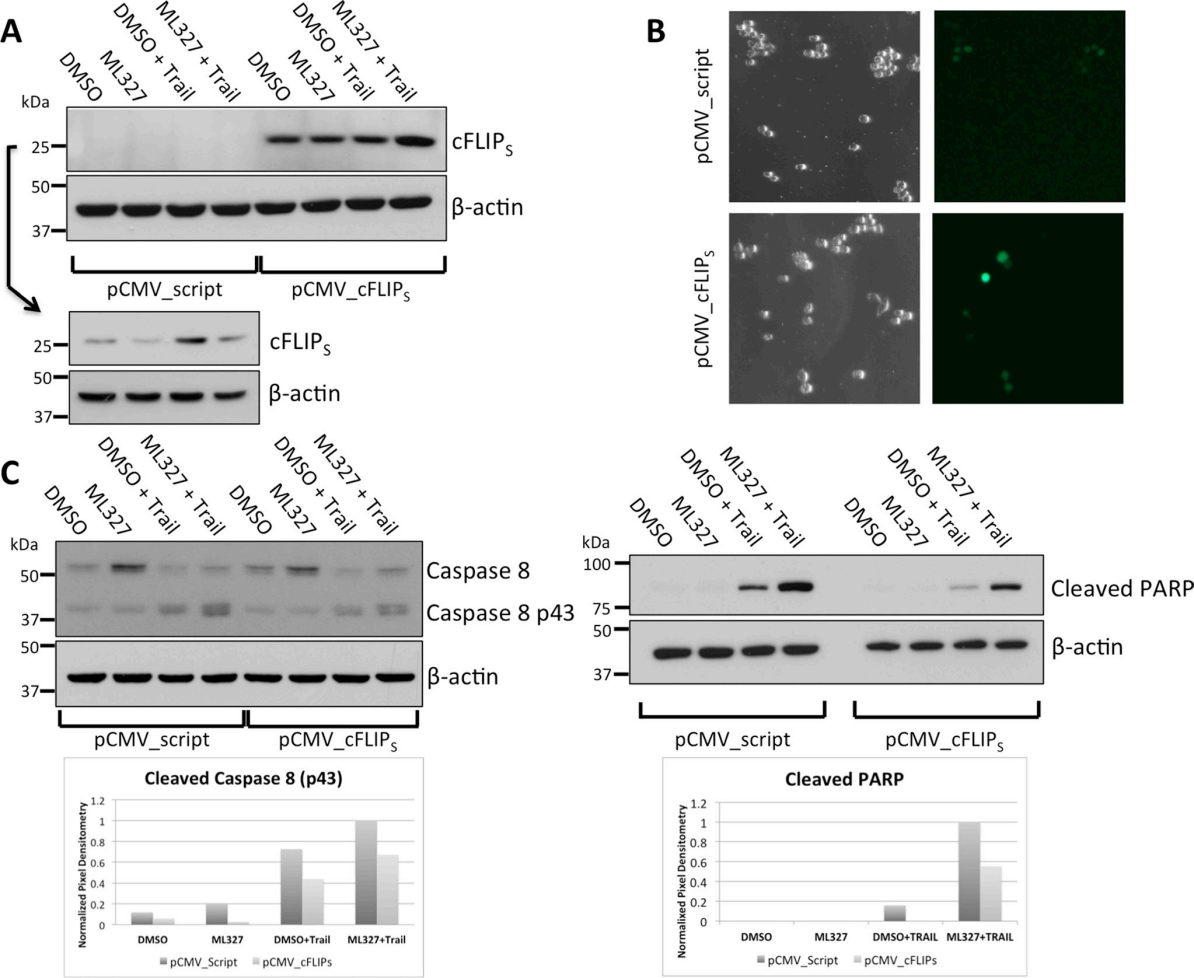
cFLIP_S_ overexpression blunts ML327-indued sensitization to TRAIL (**A**) Western blot showing cFLIP_S_ levels in SW620 cells overexpressing cFLIP_S_ or empty vector control. Due to the relatively low abundance of cFLIP_S_ in control-transfected cells, a separate gel was run to enable longer exposure for protein detection (lower panels). Representative blot of *n* = 3 biologic replicates shown. (**B**) Co-transfection with a GFP expressing plasmid was performed to assess transfection efficiency. Approximately 48 hours after transfection 10–20% of cells expressed GFP. (**C**) Western blot for caspase 8 (left panels) and cleaved PARP (right panel). 48 hours after transfection, 10 μM ML327 was added for 24 hours followed by TRAIL (100 ng/mL) for 4 hours. cFLIP_S_ over-expression resulted in an approximately 50% reduction in caspase 8 and PARP cleavage as analyzed by band pixel intensity normalized to loading control (shown in bar graph). Representative blot of *n* = 3 biologic replicates shown.

We next determined whether cFLIP_S_ loss was necessary for ML327-induced TRAIL sensitization by overexpression of exogenous cFLIP_S_. We transiently transfected a constitutively active plasmid expressing cFLIP_S_ in SW620, HCT-116, and RKO cells and showed that ML327 was unable to reduce cFLIP_S_ protein with 24-hour treatment (Figure 5A, Supplementary Figure 5A). The cells were co-transfected with a GFP-expressing plasmid to assess transfection efficiency. At 48 hours after transfection, we achieved approximately 10–30% transfection efficiency, depending on cell line (Figure 5B, Supplementary Figure 5B). Transfected cells were then pre-treated with ML327 for 24 hours followed by TRAIL for an additional 4 hours. SW620 cells with cFLIP_S_ overexpression had reduced caspase 8 and PARP cleavage (Figure 5C) after ML327 pre-treatment followed by TRAIL as compared to cells transfected with control plasmids. Similar results were seen in HCT-116 and RKO cells (Supplementary Figure 5B and 5C). Taken together, these results suggest that cFLIP_S_ protein loss is sufficient to sensitize cells to TRAIL-induced apoptosis and that cFLIP_S_ protein loss is necessary for ML327-induced TRAIL sensitization.

### cFLIP_S_ mRNA expression is reduced with EMT reversal

**Figure 6:**
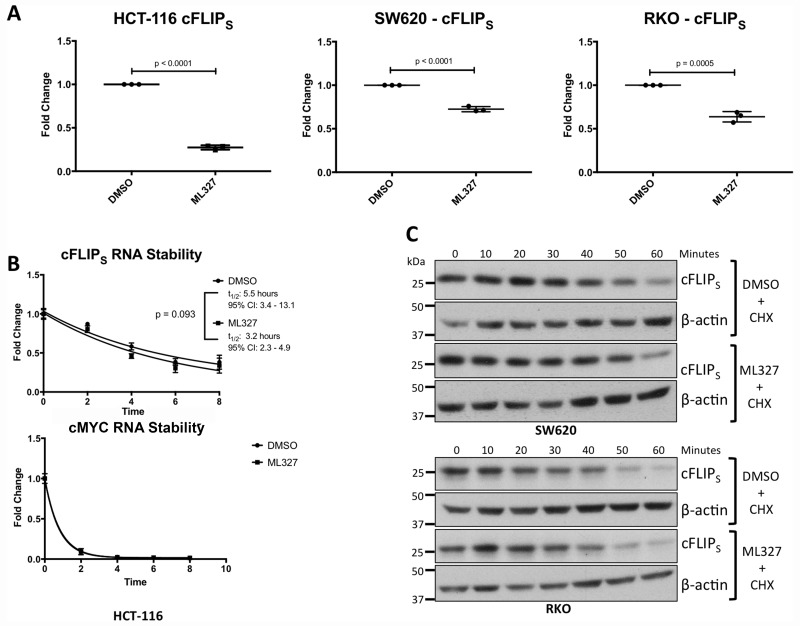
EMT reversal with ML327 causes a reduction in cFLIP_S_ mRNA expression (**A**) cFLIP_S_ mRNA levels were analyzed via RT-qPCR in 3 carcinoma cell lines after 24-hour treatment with 10 μM ML327 or vehicle. cFLIP_S_ mRNA was reduced in all 3 cancer cell lines in response to ML327 (HCT-116: *p* < 0.0001; SW620: *p* < 0.0001; RKO: *p* = 0.0005). Student *t*-test was performed to compare means. Data represented as mean ± SEM. Each data point represents a biologic replicate. Figure displays *n* = 3 biologic replicates. (**B**) cFLIP_S_ mRNA stability was assessed by pre-treating HCT-116 cells with ML327 for 2 hours followed by the addition of 5 μg/mL Actinomycin D. Cells were lysed at indicated time points and RT-qPCR was performed as above. A one phase, exponential decay analysis using a non-linear, least squares regression model was performed with no difference in cFLIP_S_ mRNA stability (*p* = 0.093) as determined by the extra sum-of-squares F test. RT-qPCR using MYC primers was performed as a positive control for actinomycin D activity. Data represented as mean ± SEM. Each data point represents a mean of *n* = 3 biologic replicates. (**C**) cFLIP_S_ protein half-life was assessed by pre-treating cells with ML327 for 6 hours followed by the addition of cycloheximide. Cells were lysed at indicated time points and western blot was performed. ML327 did not reduce cFLIP_S_ protein half-life. Figure is representative of *n* = 3 biologic replicates.

Regulation of cFLIP_S_ expression occurs at the transcriptional and post-transcriptional levels [26, 28, 32–35]. In HCT-116, SW620, and RKO cells, cFLIP_S_ mRNA levels were significantly reduced after 24-hour treatment with ML327 (HCT-116: *p* < 0.0001; SW620: *p* < 0.0001; RKO: *p* = 0.0005), mirroring the protein levels. (Figure 6A). cFLIP_L_ levels were reduced in HCT-116 cells but unchanged in SW620 and RKO cells (Supplementary Figure 6A), also mirroring protein levels.

To determine whether the effect of ML327 on cFLIP_S_ mRNA was due to differential expression of the major transcription factors known to modulate cFLIP expression, we queried our RNA sequencing data for *FOXO3*, *E2F1*, and *MYC*, NF- κB and *TP53* expression [36–40]. *FOXO3*, *E2F1*, and *MYC* (known repressors of cFLIP expression) were downregulated by ML327 treatment (Supplementary Figure 6B, Supplementary Tables 1, 2) making them unlikely mechanistic targets. On the other hand, *NFKB1* and *TP53* (known inducers of cFLIP expression) were modestly downregulated by RNA sequencing (Supplementary Figure 6B, Supplementary Tables 1, 2) but were not altered at the protein level (Supplementary Figure 6B) making these transcription factors unlikely mechanistic targets as well. Of note, *NFKB2* and *RELA* were not significantly differentially expressed after ML327 treatment in all cell lines (Supplementary Figure 6B, Supplementary Tables 1–3).

In HCT-116 cells, in which the reduction of cFLIP_S_ mRNA was greatest, mRNA stability was unchanged with ML327 treatment (Figure 6B). In SW620 and RKO cells, in which the reduction of cFLIP_S_ mRNA was not as robust, we tested for altered protein stability in response to ML327 and found none (Figure 6C). In addition, ML327 did not appear to alter global polysome profiles in SW620 and RKO cells (Supplementary Figure 6C) suggesting no differences in mRNA translation. Taken together, these results demonstrate that EMT reversal with ML327 causes a downregulation of cFLIP_S_ mRNA expression.

## DISCUSSION

Understanding the mechanism that drives EMT remains a major focus of cancer research. Small molecules that reverse EMT are useful tools as they enable manipulation of the EMT transcriptional program thereby allowing for subsequent analysis. We have previously described an isoxazole-based small molecule, ML327, that partially reverses TGF-β-induced EMT in immortalized mouse mammary epithelial cells resulting in a de-repression of E-cadherin expression [17]. In the current study, we performed RNA sequencing on 3 carcinoma cell lines after ML327 treatment and demonstrate a reversal of EMT via the downregulation of numerous EMT and stem-cell related genes. Ingenuity Pathway core expression analysis of RNA sequencing data performed on carcinoma cells treated with ML327 implicates “regulation of the EMT pathway” as a major canonical pathway. Furthermore, gene-expression profiles after ML327 treatment positively enrich with previously published gene sets associated with EMT reversal. We do note that the expression of major EMT transcription factors *TWIST1*, *SNAI1*, and *ZEB1*, is either unchanged or modestly upregulated by ML327 (Log2 FC < 1.5) in all 3 carcinoma cell lines (Supplementary Tables 1–3), suggesting that the partial EMT reversal by ML327 is disconnected from the expression of these EMT regulators through an unknown mechanism. Our data suggest that the normal function of these transcription factors as transcriptional repressors may be abrogated by ML327 as we consistently observe *CDH1* upregulation in the setting of increased *SNAI1* and *ZEB1* expression.

EMT is a major driver of apoptotic resistance in carcinomas [15] and we tested whether partial reversal with ML327 would re-sensitize carcinoma cells to TRAIL-induced apoptosis. Our data indicate that ML327 sensitizes carcinoma cells to TRAIL as evident by increases in PARP cleavage, caspase 8 cleavage, caspase 3/7 activation, Annexin V binding to PS residues, and Sub G_0_ cell population after ML327 pre-treatment. A previous report suggested that E-cadherin expression is necessary for TRAIL sensitivity [16]; however, our data support that the effect of TRAIL sensitization by ML327 is independent of E-cadherin expression. Although E-cadherin loss is considered a hallmark of EMT [3], there are many other phenotypic manifestations of this process and our data suggest that E-cadherin may be a secondary factor in EMT-associated resistance to apoptosis.

Instead of E-cadherin, our data implicate cFLIP_S_ as a critical regulator of EMT-associated resistance to apoptosis. Our results show that ML327 treatment causes downregulation of cFLIP_S_ mRNA expression with subsequent protein reduction. cFLIP_S_ knockdown by small interfering RNA was sufficient to sensitize carcinoma cells to TRAIL. While cFLIP_L_ was also knocked down, evidence suggests cFLIP_L_ is pro-apoptotic at physiologic levels [11–13] making it an unlikely regulator of ML327-induced TRAIL sensitization. Exogenous overexpression of cFLIP_S_ inhibited the ability of ML327 to sensitize carcinoma cells to TRAIL. Complete rescue was not achieved, however, and this was likely due to low transient transfection efficiencies despite multiple attempted transfection modalities. Taken together, our findings suggest that cFLIP_S_ is a critical modulator of EMT-associated resistance to apoptosis. It will be of interest to determine whether the EMT link with cFLIP_S_ also participates in the mechanism of apoptosis resistance in response to other cancer therapeutic agents.

While we have shown that ML327 sensitizes carcinoma cells to TRAIL via partial EMT reversal and downregulation of cFLIP_S_ mRNA expression, a complete understanding of the direct mechanism has not yet been elucidated. Interestingly, ML327 only consistently affects cFLIP_S_ mRNA levels and not cFLIP_L_ levels. With no appreciable difference in mRNA stability and no global difference in polysome profiles, it is possible that ML327 is modulating alternative splicing of cFLIP pre-mRNA. There are 15 known splice variants of cFLIP and while 3 are translated into proteins, the remaining 12 are targeted for degradation via the nonsense mediated decay pathway. It has been recently shown that several post-translational modifications on histones regulate alternative splicing of mRNA by modulating the recruitment of various splicing factors [41]. Unpublished data from our lab suggests that ML327 treatment causes increased H3K9 acetylation, H3K4 tri-methylation, and H3K27 acetylation on a global scale lending to the possibility that ML327 is indirectly altering pre-mRNA splicing of cFLIP through these histone modifications. Further study into this mechanism is warranted.

The direct intracellular target of ML327 has yet to be identified. Radioisotope, biotin, and iodine labeling of ML327 have all been attempted but these modifications have reduced the biological activity of ML327 *in vitro* suggesting that they reduce target affinity (data not shown). EMT reversal has been reported with other experimental therapeutics including histone deacetylase inhibitors (HDACi) and bromodomain inhibitors. Indeed, Trichostatin A, a non-specific HDACi, has been shown to derepress E-cadherin in carcinoma cells [42]. These compounds have also been shown to downregulate cFLIP, albeit by a reduction in protein stability [43, 44]. We have tested whether ML327 inhibits class I, II, III, and IV HDACs and found it to have no direct inhibitory effect on histone deacetylase activity [42]. Efforts are ongoing to determine the direct mechanism of action for ML327.

Despite an elusive intracellular target protein, ML327 remains an intriguing small molecule with therapeutic potential. *In vivo* studies have demonstrated that ML327 can be delivered intraperitoneally in a mouse model with excellent drug metabolism and pharmacokinetics (DMPK) profiles [18]. Further studies with ML327 in pre-clinical animal models are ongoing.

In conclusion, EMT is a mechanism linked to acquisition of resistance to apoptosis-inducing agents in cancer cells. This study demonstrates EMT reversal with ML327 sensitizes carcinoma cells to TRAIL-induced apoptosis and implicates cFLIP_S_ as the critical link between EMT and apoptosis resistance. While the exact mechanism of cFLIP_S_ downregulation by ML327 is still unknown, the therapeutic implications of small molecules that can reverse EMT warrant further investigation as they may provide improved understanding of therapeutic resistance in cancer along with potentially novel therapeutic approaches.

## MATERIALS AND METHODS

### Reagents

TRAIL was purchased from Bio Vision (#4354-50, San Francisco, CA) and was also graciously provided by Dr. Avi Ashkenazi (Genetech, San Francisco, CA). Actinomycin D was purchased from EMD Millipore (Billerica, MA). Cycloheximide was purchased from Sigma-Aldrich (St. Louis, MO).

### Cell culture

The HCT-116 parental cell line (HD-PAR-007) was obtained from Horizon Discovery Group (Cambridge, United Kingdom) and the SW620 (CCL-227), RKO (CRL-2577), A549 (CCL-185) and MDA-MB-231 (HTB-26) cell lines were obtained from American Type Culture Collection (ATCC) (Manassas, VA). Cells were maintained in humidified incubator at 37°C and 5% CO_2_. All cell lines were routinely cultured in RPMI 1640 media with L-glutamine (Thermo Fisher Scientific, Waltham, MA) supplemented with 10% fetal bovine serum (FBS) (Atlanta Biologicals, Norcross, GA), and 50 μg/mL of penicillin/streptomycin (Corning, Manassas, VA). Cell lines were authenticated via short tandem repeat (STR) profiling by Genetica Cell Line Testing (Burlington, NC) on a yearly basis, most recently in September, 2016. All cell lines were routinely tested for mycoplasma contamination using the MycoSensor PCR Assay Kit – #302108 – (Agilent Technologies, Santa Clara, CA) and were negative.

### RNA isolation and quantitative RT-PCR

RNA was extracted from cells using the RNeasy kits (Qiagen, Valencia, CA) per manufacturer's instructions. RNA concentrations were measured using a NanoDrop spectrophotometer (Thermo Scientific, Wilmington, DE). cDNA was reverse transcribed using iScript^™^ Reverse Transcription Supermix for RT-qPCR (Bio Rad, Hercules, CA) per manufacturer's instructions. Quantitative RT-PCR was performed using a LightCycler^®^ 480 II instrument (Roche Biologicals, Basel, Switzerland). PCR instrumentation and reagents were obtained through Roche Biologicals (Basel, Switzerland). cFLIP_S_ primers were designed as described in Ewald et al [45] and RT-qPCR was performed using the iQ SYBR^®^ Green Supermix (Bio Rad, Hercules, CA). cFLIP_S_ Forward Primer Sequence: 5′– GCAGCAATCCAAAAGAGTCTCA – 3′. cFLIP_S_ Reverse Primer Sequence: 5′ –ATTTCCAAG AATTTTCAGATCAGGA– 3′. cFLIP_L_ and cMYC primers and corresponding probe were designed using the Universal ProbeLibrary Assay Design Center (Roche Biologicals) and RT-qPCR was performed using the monocolor hydrolysis probe/UPL probe system (Roche Biologicals). cFLIP_L_ Forward Primer Sequence: 5′ – GCT CACCATCCCTGTACCTG – 3′. cFLIP_L_ Reverse Primer Sequence: 5′ – CAGGAGTGGGCGTTTTCTT – 3′. Associated probe for the cFLIP_L_ primers was UPL # 14. MYC Forward Primer Sequence: 5′ – GCTGCTTAG ACGCTGGATTT – 3′. MYC Reverse Primer Sequence: 5′ – TAACGTTGAGGGGCATCG – 3′. Associated probe for the cMYC primers was UPL # 66.

### RNA sequencing

RNA from HCT-116, SW620, and A549 cancer cells (*n* = 3 per group) treated with either DMSO or ML327 for 24 hours was collected using RNeasy kits. Processing of RNA using a TruSeq Stranded mRNA sample prep kit was conducted according to the manufacturer's instructions (Illumina, San Diego, CA). Approximately 27-36 million 50 base pair single-end reads were generated, per sample. We mapped the reads to the human genome hg19 using TopHat-2.0.10 [46]. 96% of the reads were mapped to the genome. Then, following the method of Anders et al [47], we counted the number of reads that fell into annotated genes by samtools-0.1.19 [48] and HTSeq-0.5.4p5 [49]. Finally, we performed count-based differential expression analysis using edgeR_3.4.2 [50], which implements general differential analyses based on the negative binomial model.

### Gene set enrichment analysis and ingenuity pathway analysis

Gene set enrichment analysis software was obtained through the Broad Institute (http://software.broadinstitute.org/gsea/index.jsp) and analyses were performed according to Broad Institute guidelines [51, 52]. Gene sets were obtained from the Molecular Signatures Database (MSigDB) 4.0 (http://www.broadinstitute.org/gsea/msigdb/). Ingenuity Pathway Analysis software was obtained from QIAGEN Bioinformatics (QIAGEN, Redwood City, CA). Differentially expressed gene lists were analyzed according to software specifications. Canonical pathway analysis was performed as described in Haddad et al. [53].

### Protein expression

Whole cells were lysed in RIPA buffer (150 mM NaCl, 1% NP-40, 0.5% Na deoxycholate, 0.1% SDS, 50 mM Tris-Cl, pH = 8.0) supplemented with a protease inhibitor cocktail consisting of 1 μg/mL aprotinin, 1 μg/mL leupeptin, 3 μg/mL Pepstatin, 1 mM NaVO3, 1 mM NaF, 0.5 μM DTT (Sigma Chemical, St. Louis, MO). Protein lysates were separated on 10% SDS-polyacrylamide gels. Antibodies used were as follows: cFLIP human (Enzo Life Sciences #ALX-804-961-0100, Farmingdale, NY) [16]; cFLIP mouse (Abcam #ab8421, Cambridge, MA); total PARP, cleaved PARP, caspase 8, TRAIL-R2, p53, and NFKB1 (Cell Signaling Technology #9532, #5625, #9746, #8074, #2524, and #13586 Danvers, MA); TRAIL-R1 (Thermo Scientific, #32A1380); E-cadherin (BD Transduction Laboratories #610182, Franklin Lakes, NJ); β-actin (Sigma Chemical #A5441, St. Louis, MO); GAPDH (Life Technologies #AM4300, Carlsbad, CA); anti-mouse and anti-rabbit secondary antibodies (Santa Cruz Biotechnology #sc2005 and #sc-2004, Dallas, TX). Chemiluminescent western blot analyses were performed using ECL detection (EMD Millipore, Darmstadt, Germany).

### Cell cycle analysis

Cells were trypsinized and fixed in 70% ethanol overnight at 4^°^C. Fixed cells were re-suspended in phosphate buffered saline (PBS) and stained with a propidium iodide (PI) cocktail consisting of PI, ribonuclease, and PBS. FACS cell-cycle analysis was performed after 30-minute incubation. Flow Cytometry experiments were performed on a 5-Laser BD LSRII in the Vanderbilt University Medical Center (VUMC) Flow Cytometry Shared Resource.

### Caspase 3/7 activity assay

Caspase 3/7 activity assay was performed using Caspase-Glo^®^ 3/7 Assay System (Promega Corporation, Madison, WI) according to manufacturer instructions. Cells were grown in Corning 96-well, clear bottom, white polystyrene plates (Corning, NY) at an approximate density of 15,000 cells/well. At the conclusion of the experiment, luminescence was measured on a SpectraMax i3 Multi-Mode Platform (Molecular Devices, Sunnyvale, CA).

### Annexin V assay

Annexin V assay was performed using the RealTime-Glo^TM^ Annexin V Apoptosis Assay (Promega Corporation, Madison, WI) according to manufacturer instructions. Cells were grown in Corning 96-well, clear bottom, white polystyrene plates (Corning, NY) at an approximate density of 15,000 cells/well. Hourly luminescence measurements were measured on a SpectraMax i3 Multi-Mode Platform (Molecular Devices, Sunnyvale, CA).

### Gene knockdown

siRNA knockdown was performed using pooled siRNA oligonucleotides obtained from Dharmacon^™^ (Lafayette, CO). Sequences are as follows: si-CFLAR 1: 5′ GUGCCGGGAUGUUGCUAUA 3′ si-CFLAR 2: 5′ CAA GCAGUCUGUUCAAGGA 3′ si-CFLAR 3: 5′ CAUGGU AUAUCCCAGAUUC 3′ si-CFLAR 4: 5′ CCUAGGAAU CUGCCUGAUA 3′ si-CDH1 1: 5′ GGCCUGAAGUGA CUCGUAA 3′ si-CDH1 2: 5′ GAGAACGCAUUGCCAC AUA 3′ si-CDH1 3: 5’ GGGACAACGUUUAUUACUA 3′ si-CDH1 4: 5′GACAAUGGUUCUCCAGUUG 3′ Cells were transfected using Dharmacon^™^ DharmaFECT^™^ transfection reagent according to manufacturer's guidelines. Non-targeting siRNA, also obtained from Dharmacon^™^ were used as controls.

### Gene overexpression

cFLIP short (Transcript Variant 3) plasmid was purchased from OriGene (Rockville, MD), amplified in *E. coli*, and purified using the QIAGEN Maxi Kit (Qiagen, Valencia, CA) per manufacturer's instructions. DNA was sequenced (GeneWiz, South Plainfield, NJ) using sequencing primers purchased from OriGene and sequences were validated using NCBI Basic Local Alignment Search Tool (https://blast.ncbi.nlm.nih.gov/Blast.cgi). Cells were transfected with plasmid DNA using Effectene transfection reagent (Qiagen) per manufacturer's instructions. Transfection efficiency was determined by co-transfecting a GFP expressing plasmid and fluorescent microscopic analysis.

### Polysome profiling

### Buffers

Polysome lysis buffer was prepared as follows: 10 mM Tris-Cl, pH:7.5; 100 mM NaCl; 30 mM MgCl_2_; 200 μg/mL Heparin, 100 μg/mL Cycloheximide; 0.5% Triton X-100. Polysome gradient buffer (10×) was prepared as follows: 500 mM Tris-Cl, pH:7; 500 mM NH_4_Cl, 120 mM MgCl_2_. 50% and 10% sucrose solutions were prepared from appropriate volumes of 70% stock sucrose solution and 10× gradient buffer and cycloheximide was added at a 50 μg/mL concentration.

### Analysis

Cells were treated *in-vitro* with ML327 (or vehicle control) for 24 hours and then cycloheximide (100 μg/mL) was added for 5 minutes. Cells were then lysed in polysome lysis buffer. Lysates were centrifuged at 1000g for 3 minutes at 4^°^C. OD260 of the cleared lysate was measured on the NanoDrop (Thermo Scientific, Wilmington, DE). 15 OD units of lysate were layered onto 13 mL 50%–10% sucrose gradients. Gradients were then centrifuged using a Beckman SW-41 rotor at 222,000 g for 3 hours at 4°C. Polysome profiling was then performed as previously described by Link et al [54].

### Statistical analysis

All *in vitro* experiments were performed at least three separate times (except for caspase 3/7 and Annexin V luminescence experiments) to ensure validity of results. For all *in vitro* experiments, means were compared with the student t-test, one-way ANOVA with Tukey correction for multiple comparisons (≥ 3 comparisons), or two-way ANOVA with Tukey correction for multiple comparisons (≥ 3 comparisons).

## SUPPLEMENTARY MATERIALS FIGURES AND TABLES











## Abbreviations

EMT: epithelial to mesenchymal transition
cFLIP: cellular FLICE-like Inhibitory Protein
TRAIL: tumor necrosis factor-related apoptosis inducing ligand
PARP: poly ADP-ribose polymerase
PS: phosphatidylserine
HDAC: histone deacetylase
DMPK: drug metabolism and pharmacokinetics.
