# Supplementary material for: cFLIP critically modulates apoptotic resistance in epithelial-to-mesenchymal transition
Source: Oncotarget. 2017 Jul 25;8(60):101072–86. doi: 10.18632/oncotarget.19557 (PMC5731856; doi:10.18632/oncotarget.19557)
Supplement: Supplementary file 1 [file oncotarget-08-101072-s001.pdf]

cFLIP critically modulates apoptotic resistance in epithelial-to-mesenchymal transition

SUPPLEMENTARY MATERIALS

A

SW620

Ingenuity Canonical Pathways	p-value
Human Embryonic Stem Cell Pluripotency	2.45E-10
Axonal Guidance Signaling	7.76E-10
Role of NANOG in Mammalian Embryonic Stem Cell Pluripotency	3.63E-09
PCP pathway	6.03E-08
**Regulation of the Epithelial-Mesenchymal Transition Pathway**	**1.38E-06**
Wnt/Ca+ pathway	8.71E-05
Wnt/β-catenin Signaling	1.26E-04
Factors Promoting Cardiogenesis in Vertebrates	6.03E-04
Sonic Hedgehog Signaling	9.33E-04
Notch Signaling	2.75E-03

HCT116

Ingenuity Canonical Pathways	p-value
Axonal Guidance Signaling	7.41E-08
Human Embryonic Stem Cell Pluripotency	1.48E-03
Factors Promoting Cardiogenesis in Vertebrates	1.66E-03
NF-κB Signaling	3.02E-03
Wnt/β-catenin Signaling	7.76E-03
Notch Signaling	1.26E-02
PCP pathway	1.74E-02
Role of NANOG in Mammalian Embryonic Stem Cell Pluripotency	1.78E-02
Sperm Motility	2.69E-02
**Regulation of the Epithelial-Mesenchymal Transition Pathway**	**3.39E-02**

A549

Ingenuity Canonical Pathways	p-value
Axonal Guidance Signaling	5.01E-15
Netrin Signaling	3.80E-05
Human Embryonic Stem Cell Pluripotency	9.33E-05
Ephrin Receptor Signaling	1.15E-04
PCP pathway	2.57E-04
Wnt/β-catenin Signaling	1.07E-03
**Regulation of the Epithelial-Mesenchymal Transition Pathway**	**3.24E-03**
Inhibition of Matrix Metalloproteases	3.98E-03
Role of NANOG in Mammalian Embryonic Stem Cell Pluripotency	7.76E-03
Sperm Motility	1.66E-02

B

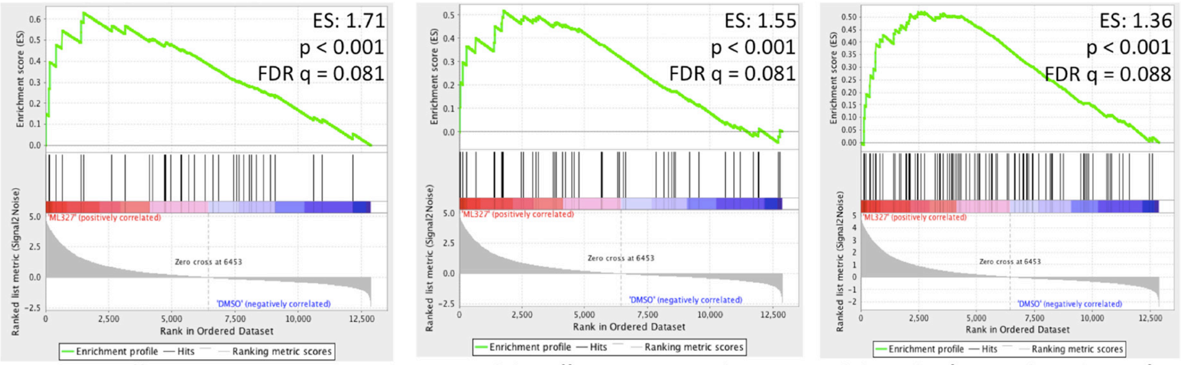

GO Adherens Junction

GO Adherens Junction Organization

GO Apical Junction Complex

Supplementary Figure 1: (**A**) Canonical pathway analysis of RNA sequencing data in Ingenuity Pathway Analysis® demonstrates "Regulation of EMT Pathway" as one of the top 10 organismal growth and development pathways in SW620 ($p = 1.38 \times 10^{-6}$), HCT-116 ($p = 3.39 \times 10^{-2}$), and A549 ($p = 3.24 \times 10^{-3}$) cells. Additional top pathways are also shown. (**B**) Additional GSEAs performed demonstrating signatures associated with GO adherens junction organization and function (GO Version 5.2).

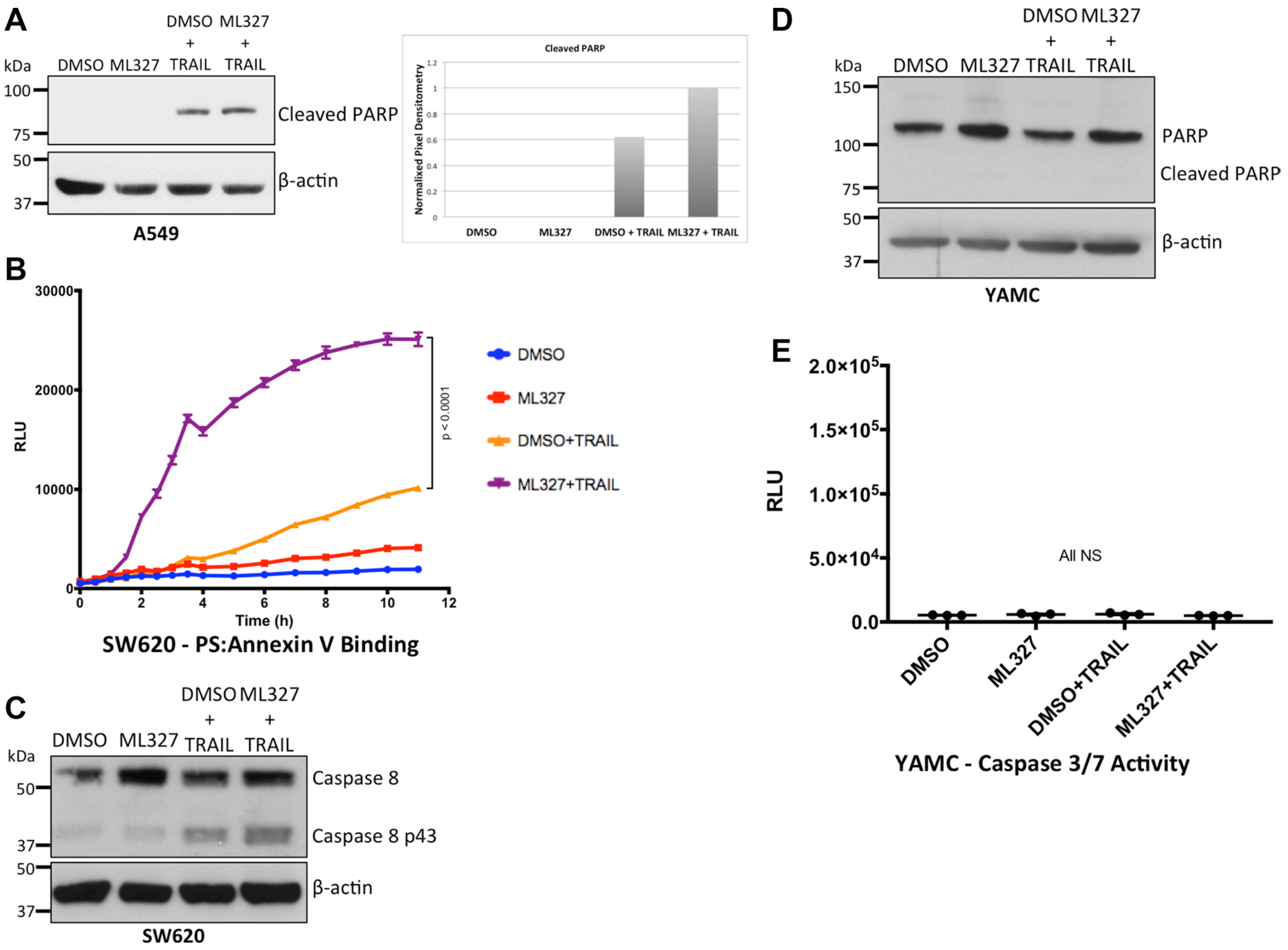

Supplementary Figure 2: (**A**) Western blot for cleaved PARP shown in A549 cells treated with 10 µM ML327 (or vehicle) for 24 hours followed by TRAIL (200 ng/mL). ML327 pre-treatment resulted in increased PARP cleavage as compared to vehicle pre-treatment. Cleaved PARP band densitometry normalized to β-actin loading controls shown in bar graph. (**B**) RealTime™ Glo Annexin V assay was performed in SW620 cells pre-treated with 10 µM ML327 (or vehicle) for 24 hours followed by TRAIL (100 ng/mL). Hourly luminescence measurements were performed demonstrating increased Annexin V binding to phosphatidylserine (PS) residues ($P < 0.0001$) in the ML327 pre-treated cells. Data presented as mean ± SEM of 4 technical replicates. Area under the curve analysis performed to compare curves. (**C**) Western blot for caspase 8 shown in SW620 colon cancer cell lines treated with 10 mM ML327 (or vehicle) for 24 hours followed by TRAIL (100 ng/mL). ML327 pre-treatment resulted in increased caspase 8 cleavage. Figure is representative of $n = 3$ biologic replicates. (**D**) Western blot for PARP shown in YAMC cells pre-treated with 10 µM ML327 (or vehicle) for 24 hours followed by TRAIL (100 ng/mL). Western blot analysis shows no PARP cleavage with TRAIL alone or with ML327 pre-treatment. This experiment was performed concurrently with those in Figure 2 as positive controls. (**E**) Caspase Glo® 3/7 assay was performed on YAMC cells treated as in (b) and there was no difference from baseline caspase 3/7 activity (all $p > 0.24$) as measured by luminescence (RLU). This experiment was performed concurrently with those in Figure 2 as positive controls. Data represented as mean ± SEM. Data points represent technical replicates. One-way ANOVA was performed to compare all means.

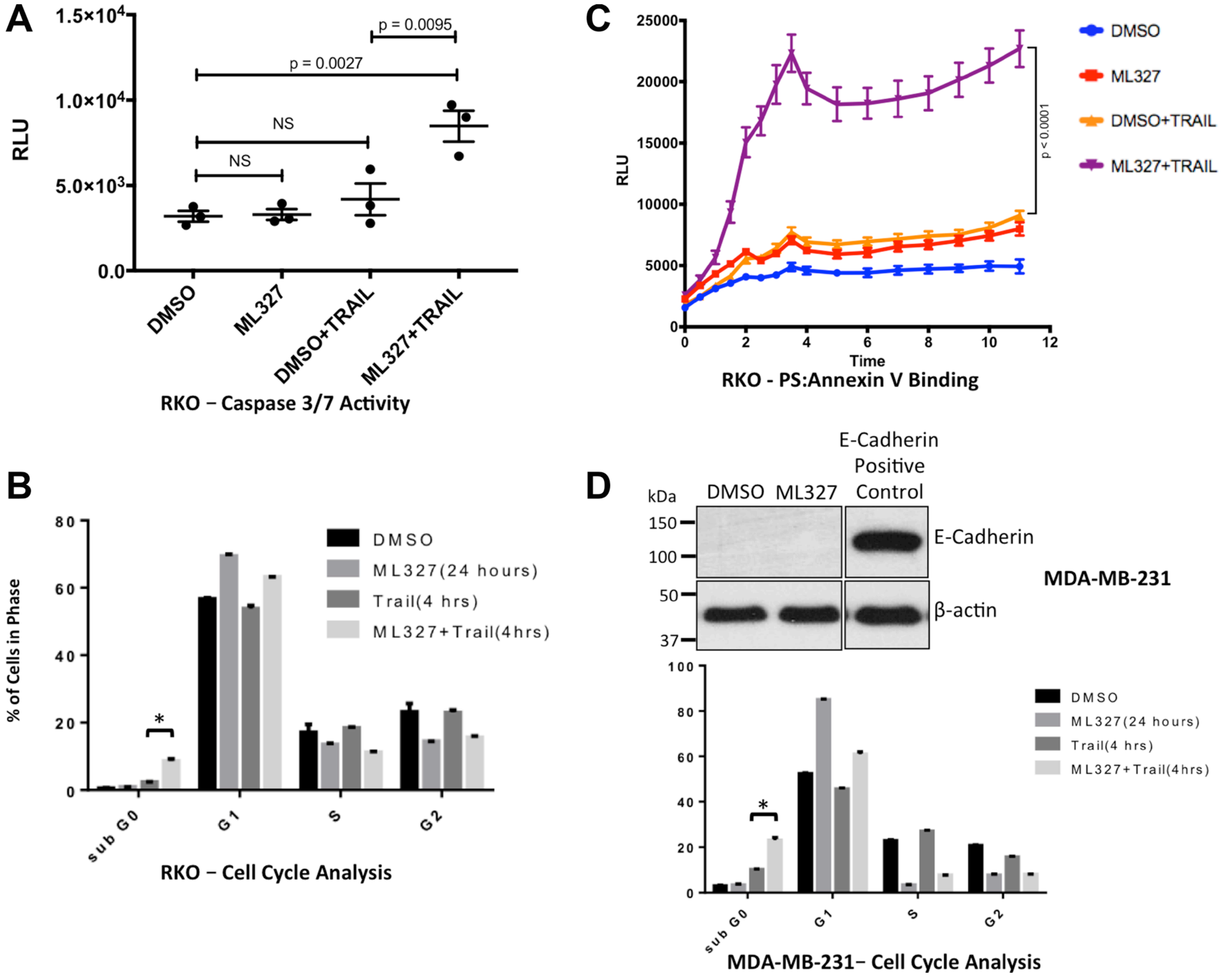

Supplementary Figure 3: (**A**) Caspase Glo® 3/7 assay performed on RKO cells pre-treated with 10 µM ML327 or vehicle for 24 hours followed by 500 ng/mL TRAIL for 4 hours. Cells pre-treated with ML327 followed by TRAIL demonstrated increased caspase 3/7 activity as measured by luminescence (RLU) compared to vehicle pre-treatment ($p = 0.0095$). Data presented as mean ± SEM. Data points represent technical replicates. One-way ANOVA was performed to compare all means. (**B**) Flow cytometric cell cycle analysis performed on RKO cells pre-treated with 10 µM ML327 (or vehicle) for 24 hours followed by TRAIL (500 ng/mL) for 4 hours. ML327 pre-treatment followed by TRAIL resulted in increased population of cells in the Sub-G_0 phase as compared to vehicle pre-treatment ($p < 0.001$). (**C**) RealTime™ Glo Annexin V assay was performed in RKO cells pre-treated with 10 µM ML327 (or vehicle) for 24 hours followed by TRAIL (500 ng/mL). Hourly luminescence measurements were performed demonstrating increased Annexin V binding to PS residues ($p < 0.0001$) in the ML327 pre-treated cells. Data presented as mean ± SEM of 4 technical replicates. Area under the curve analysis performed to compare curves. (**D**) Western blot for E-cadherin and flow cytometric cell cycle analysis shown in MDA-MB-231 cells. These cells do not re-express E-cadherin with ML327 treatment. E-cadherin positive control utilized was the same concentration of protein lysate derived from HCT-116 cells. Cell cycle analysis performed after pre-treatment with 10 µM ML327 (or vehicle) for 24 hours followed by TRAIL (100 ng/mL) for 4 hours resulted in increased population of cells in the Sub-G_0 phase as compared to vehicle pre-treatment.

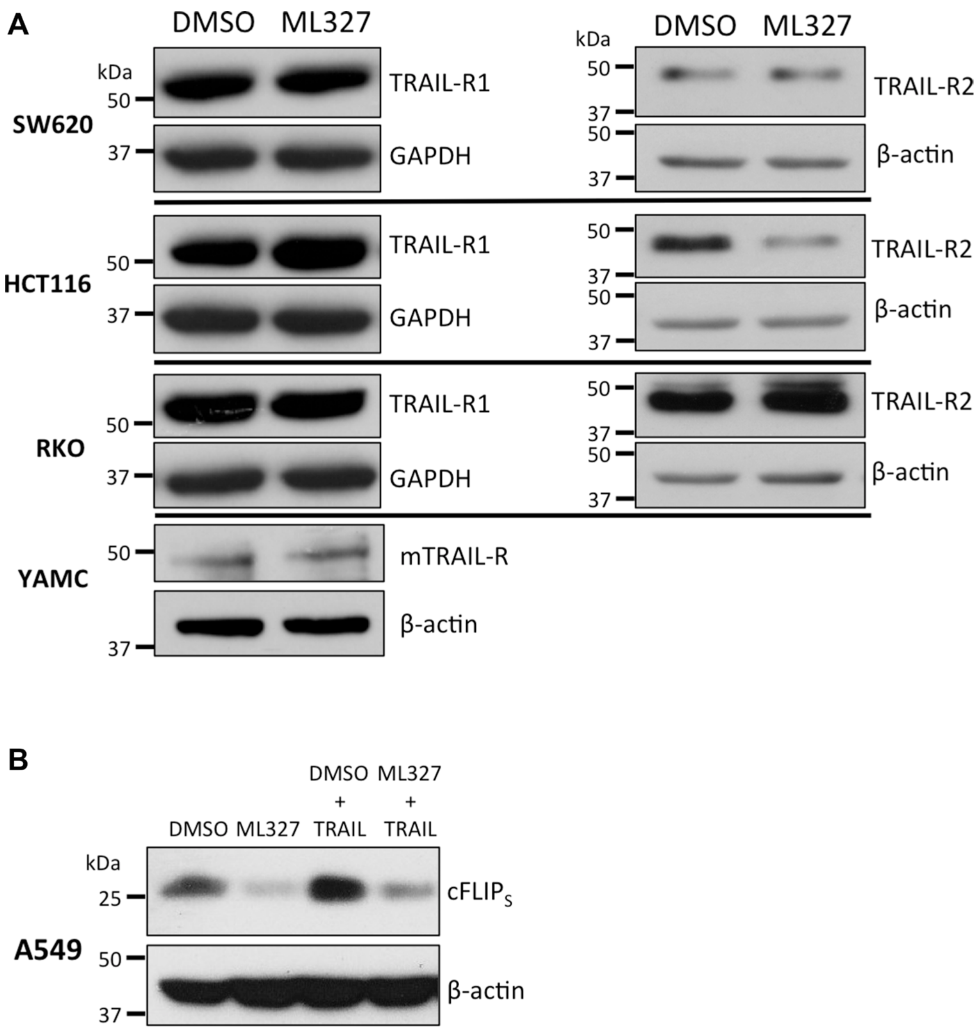

Supplementary Figure 4: (**A**) Western blots showing human TRAIL-R1 and TRAIL-R2 as well as mouse mTRAIL-R expression with 24 hour, 10 µM ML327 (or vehicle) treatment. ML327 did not alter TRAIL-R1 and mTRAIL-R expression. TRAIL-R2 expression was reduced in HCT-116 cells and unchanged in SW620 and RKO cells. TRAIL receptor protein band migration was not shifted with ML327 treatment suggesting no alteration in glycosylation states. (**B**) Western blot showing cFLIP$_S$ down-regulation in A549 cells after 24 hour, 10 µM ML327 treatment with and without TRAIL (200 ng/mL).

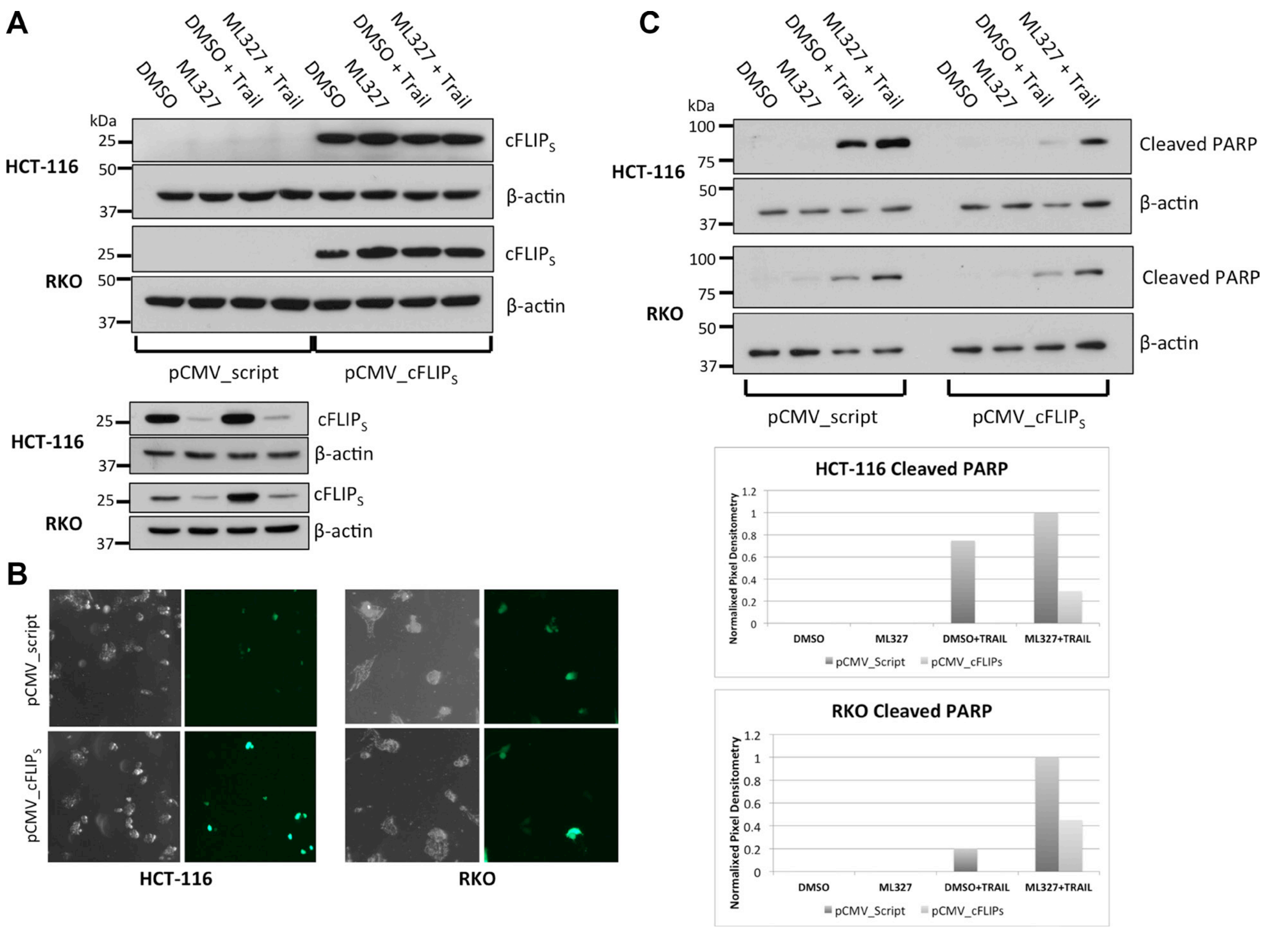

Supplementary Figure 5: (**A**) Western blot showing cFLIP$_S$ levels in HCT116 and RKO cells overexpressing cFLIP$_S$ or empty vector control. Due to the relatively low abundance of cFLIP$_S$ in control-transfected cells, a separate gel was run to enable longer exposure for protein detection (lower panels). (**B**) Co-transfection with a GFP expressing plasmid was performed to assess transfection efficiency. Approximately 48 hours after transfection 20–30% of HCT-116 and RKO cells expressed GFP. (**C**) Western blot for cleaved PARP. 48 hours after transfection, 10 μM ML327 was added for 24 hours followed by TRAIL (HCT-116: 50 ng/mL, RKO: 500 ng/mL) for 4 hours. cFLIP$_S$ over-expression resulted in an approximately 50% reduction in PARP cleavage as analyzed by band pixel intensity normalized to loading control (shown in bar graphs). Figures are representative of n = 3 biologic replicates.

A

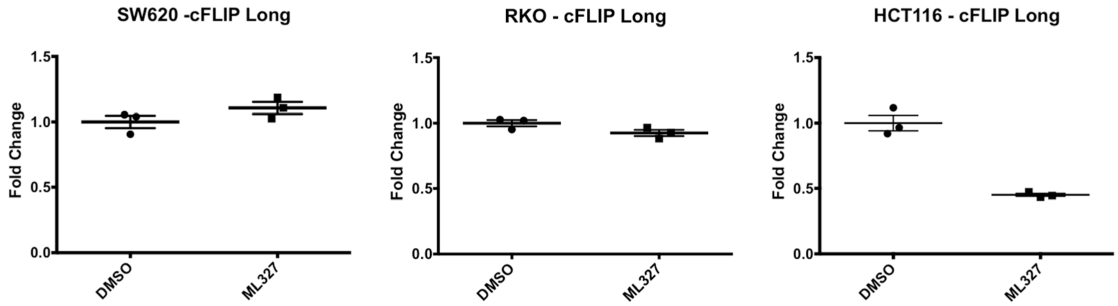

B

		RNA Sequencing Result					
		HCT116			SW620		
Transcription Factor	Published Effect on cFLIP	Log FC	P Value	FDR	Log FC	P Value	FDR
E2F1	Downregulates	-0.0338601	0.79392041	0.85534943	-0.1578903	0.13061385	0.17870921
FOXO3	Downregulates	-0.2240203	0.13722183	0.23011903	-0.3992236	1.39E-05	4.04E-05
MYC	Downregulates	-1.0669212	2.19E-09	2.36E-08	-0.0356601	0.69572031	0.74919075
NFKB1	Upregulates	-0.8708865	2.43E-13	4.03E-12	-0.4544459	1.43E-07	5.22E-07
NFKB2	Upregulates	-0.0093746	0.94011779	0.96050291	0.52460309	4.00E-06	1.24E-05
RELA	Upregulates	-0.0536994	0.6557379	0.75018645	-0.1668535	0.07737468	0.11263737
TP53	Upregulates	-0.8781104	3.10E-13	5.09E-12	-0.4712547	6.88E-09	2.85E-08

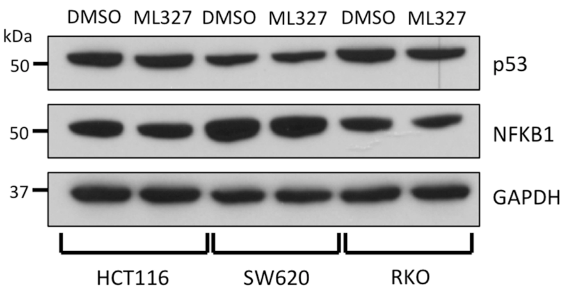

C

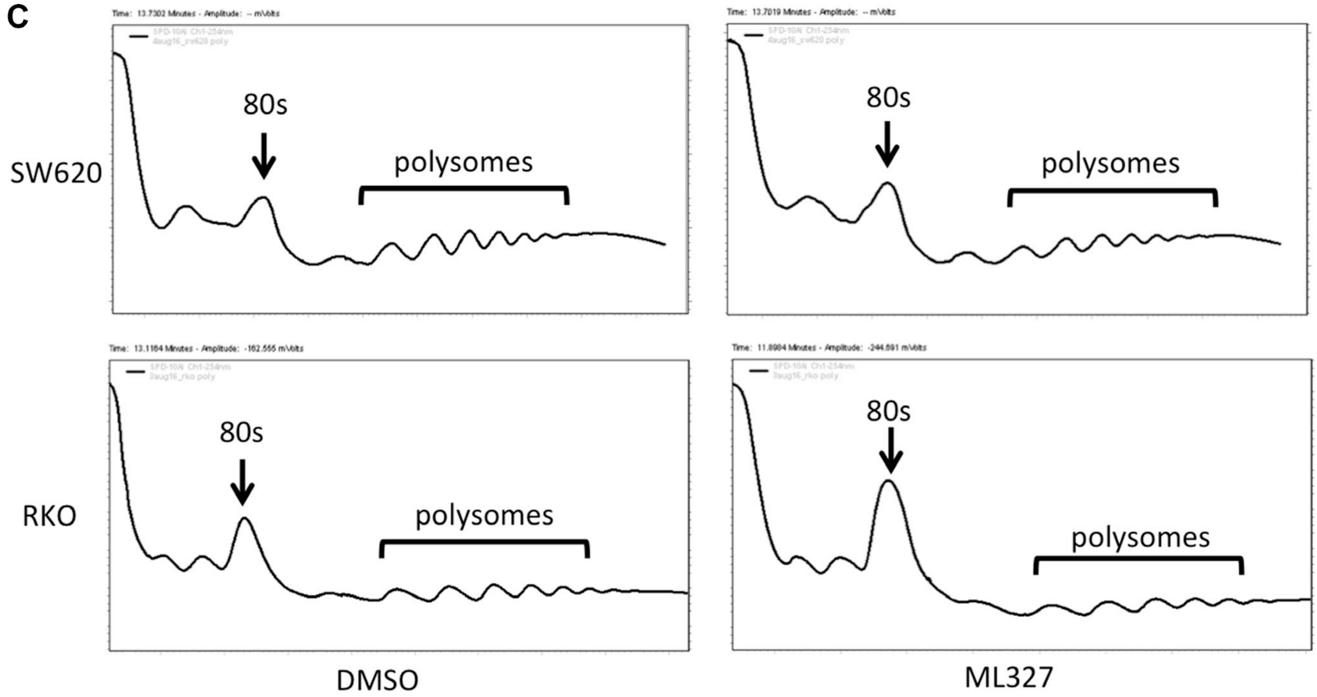

Supplementary Figure 6: (**A**) cFLIP$_L$ mRNA levels were not consistently reduced with ML327 treatment by RT-qPCR. (**B**) Summary table and subsequent western blot validation of differentially expressed transcription factors known to modulate cFLIP expression. *E2F1*, *FOXO3*, and *MYC* – known repressors of cFLIP expression – are downregulated by ML327 treatment making them unlikely targets of ML327. *NFKB1* and *TP53* – known inducers of cFLIP expression – are downregulated by RNA sequencing but no difference in protein level is detected on western blot. *NFKB2* and *RELA* are not significantly differentially expressed on RNA sequencing. (**C**) Polysomal mRNA analysis performed in SW620 and RKO cell lines demonstrated no change in polysomal absorbance with ML327.

Supplementary Table 1: RNA sequencing raw data for HCT-116 cell line treated with 10 mM ML327 (or vehicle control) for 24 hours. See Supplementary_Table_1.

Supplementary Table 2: RNA sequencing raw data for SW620 cell line treated with 10 mM ML327 (or vehicle control) for 24 hrs. See Supplementary_Table_2.

Supplementary Table 3: RNA sequencing raw data for SW620 cell line treated with 10 mM ML327 (or vehicle control) for 24 hrs. See Supplementary_Table_3.

Supplementary Table 4: 730 commonly upregulated (Log2 FC > 1.5, FDR < 0.0001) and 37 downregulated (Log2 FC < 0.667, FDR < 0.0001) across SW620, HCT-116, and A549 cells treated with 10 μM ML327 for 24 hours. See Supplementary_Table_4.
